# Gemcitabine resistance of pancreatic cancer cells is mediated by IGF1R dependent upregulation of CD44 expression and isoform switching

**DOI:** 10.1038/s41419-022-05103-1

**Published:** 2022-08-05

**Authors:** Chen Chen, Shujie Zhao, Xiangru Zhao, Lin Cao, Anand Karnad, Addanki P. Kumar, James W. Freeman

**Affiliations:** 1 Key Laboratory of Carbohydrate Chemistry and Biotechnology, Ministry of Education, School of Life Science and Health Engineering, Jiangnan University, 214122 Wuxi, P. R. China; 2 Department of Medicine, Division of Hematology and Oncology, University of Texas Health, San Antonio, TX USA; 3 Mays Cancer Center at UT Health San Antonio-MD Anderson Cancer Center, San Antonio, TX USA; 4 Department of Urology, University of Texas Health Science Center at San Antonio, San Antonio, TX USA; 5 Research and Development, Audie Murphy Veterans Administration Hospital, San Antonio, TX USA

**Keywords:** Chemotherapy, Drug development

## Abstract

Chemoresistance in pancreatic cancer cells may be caused by the expansion of inherently resistant cancer cells or by the adaptive plasticity of initially sensitive cancer cells. We investigated how CD44 isoforms switching contributed to gemcitabine resistance. Treating CD44 null/low single-cell clones with increasing amounts of gemcitabine caused an increase in expression of CD44 and development of gemcitabine resistant (GR) cells. Drug sensitivity, invasiveness, and EMT process was evaluated by MTT, Matrigel invasion assays, and western blots. Genetic knockdown and pharmacological inhibitors were used to examine the roles of CD44 and IGF1R in mediating gemcitabine resistance. CD44 promoter activity and its interactive EMT-related transcription factors were evaluated by luciferase reporter assay and chromatin immunoprecipitation assay. Kaplan–Meier curve was created by log-rank test to reveal the clinical relevance of CD44 and IGF1R expression in patients. We found silence of CD44 in GR cells partially restored E-cadherin expression, reduced ZEB1 expression, and increased drug sensitivity. The gemcitabine-induced CD44 expressing and isoform switching were associated with an increase in nuclear accumulation of phosphor-cJun, Ets1, and Egr1 and binding of these transcription factors to the CD44 promoter. Gemcitabine treatment induced phosphorylation of IGF1R and increased the expression of phosphor-cJun, Ets1, and Egr1 within 72 h. Stimulation or suppression of IGF1R signaling or its downstream target promoted or blocked CD44 promoter activity. Clinically, patients whose tumors expressed high levels of CD44/IGF1R showed a poor prognosis. This study suggests that IGF1R-dependent CD44 isoform switching confers pancreatic cancer cells to undergo an adaptive change in response to gemcitabine and provides the basis for improved targeted therapy of pancreatic cancer.

## Introduction

Pancreatic ductal adenocarcinomas (PDAC) is the fourth leading cause of cancer death in the US and has the worst prognosis of all solid tumors [1–3]. Alarmingly, total deaths from pancreatic cancer are increasing with a prediction of being the second greatest cause of cancer deaths by 2030 [4]. Although patients with PDAC may show an initial response to chemotherapy, they generally develop resistance during their course of therapy [5]. The mechanisms by which these cancer cells escape chemotherapy include the expansion of an existing population of chemo-resistant cancer cells [6, 7], the development of secondary mutations that cause resistance [6, 8, 9] or the expression of genes that switch the phenotypes of cancer cells to one that is better able to survive [10, 11].

The capacity of cells to change phenotypes in response to environmental stimuli is known as adaptive plasticity [12, 13]. An example of adaptive plasticity is the ability of epithelial cells to undergo a switch from an epithelial to a mesenchymal phenotype. Embryonic epithelial cells can undergo an epithelial to mesenchymal transition (EMT) enabling them to become migratory and move to distant regions and then undergo a mesenchymal to epithelial transition (MET) to form colonization in tissues and organs [14, 15]. A similar process named wound healing is hijacked by the cancer cells [16]. In epithelial-derived cancer cells, EMT is associated with increased cell motility, invasion, and metastases and with the acquisition of stem cell-like properties providing cells with the capacity to initiate new tumor formation [17]. Studies indicate that EMT is required for tumor spread and that MET is required for colonization and establishment of metastatic lesions [15, 16].

Others and we found that CD44 acts as a regulator of EMT and epithelial plasticity in breast, pancreatic and other cancers [18–21]. CD44 is a non-kinase transmembrane receptor that binds to hyaluronan, a proteoglycan secreted by stromal cells in response to interactions with tumor cells, and is thought to play an important role in cancer progression by activating cell signaling pathways and by modulating cytoskeletal changes favoring motility [22, 23]. CD44 is expressed in multiple isoforms through alternative splicing with the shortest of these, CD44s encoded by 10 exons and a number of CD44v expressing various combinations of an additional 10 exons [24]. The additional exons found in CD44v provide binding sites for various adhesion and signaling molecules [23, 25–27]. CD44v is involved in the colonization of metastatic cells and tumorigenicity in various cancer types [28–31]. CD44s and various CD44v isoforms have overlapping and distinct functional roles that CD44v isoforms can interact with growth factors and inflammatory cytokines expressed by tumor-associated macrophages to activate EGFR/Ras/MAPK and phospho-STAT3 signaling pathways in the tumor microenvironment [32] because of its additional binding motifs. Upstream growth factors also can bind/sequester the CD44v isoforms on the cell surface. Cancer cells with an EMT phenotype acquire stem cell-like properties and detected high CD44 expression [17]. Moreover, our lab showed that EMT-like pancreatic cancer cells become more invasive and chemo-resistant [21]. CD44 is recognized as a marker for cancer stem cells (CSCs) [19, 33, 34]. However, all CD44-expressing tumor cells are likely not CSCs nor do all CD44-expressing cells possess an EMT phenotype. In a recent study, Wang and colleagues found multiple subtypes of CSCs in breast cancer with some possessing a mesenchymal phenotype and others being more epithelial-like further indicating different pathways were activated in these subtypes [35]. The different functional roles of CD44 isoforms in relation to cancer development and progression are under investigation. A switch from CD44v to CD44s is required for EMT in breast cancer cell line models although the functional significance of CD44 in maintaining EMT was not established [18]. In this regard, we showed that cells expressing high levels of CD44s are more invasive and rapidly become resistant to gemcitabine when implanted into the pancreas of nude mice; however, recovery of the resistant tumors revealed that they undergo further switching of CD44 isoforms [21]. A more recent study showed that CD44 isoform switching gave rise to invasion and metastases in luminal breast carcinomas with CD44s high expressing cells responsible for migration [20]. However, the mechanisms of how cancer cells undergo CD44 isoform switching and become resistant to chemotherapy therapy are unclear.

In this study, we investigated the adaptive plasticity of pancreatic cancer cells in response to chemotherapy. We further sought to determine whether the development of gemcitabine resistance was solely due to the outgrowth of the inherently resistant CSC population or could be induced from a population of cells that are initially sensitive to gemcitabine. Studies presented here indicate that chemotherapy induces a phenotypic switch resulting in a decrease in response to chemotherapy and increasing invasiveness. These data suggest that isoform switching of CD44v to CD44s and that high levels of CD44s may in part serve to mediate protective cellular plasticity in response to chemotherapy. The possibility of CD44 isoforms switching back and forth has an important implication for understanding the biology of tumor plasticity so as to decisions for the patients’ therapy. Understanding tumor cell plasticity and how it allows cancer cells to escape therapy is a crucial step in the design of new therapeutic strategies to improve patients’ overall survival rate. One strategy will be to investigate whether chemotherapy-induced switch towards high CD44s-expressing cells can be blocked or reversed. Such a strategy would increase response to chemotherapy. The results of these studies may provide a new paradigm for design in strategies for therapy of PDAC.

## Results

### Gemcitabine treatment induces CD44 expression, isoform switching, and EMT

We previously isolated pancreatic cancer cells based on CD44 differential expression levels [21]. We found that CD44 high-expressing pancreatic cancer cells show predominantly CD44s isoform with an EMT phenotype, were highly invasive, and rapidly developed resistance to gemcitabine in vivo. It is not clear whether the development of CD44 high-expressing gemcitabine-resistant cancer cells is due to the selection of a subpopulation of already resistant cells and/or an adaption of cells in response to chemotherapy.

To distinguish intrinsic resistant cells from the acquired resistant cells, we established and expanded single-cell clones from CD44 null/low cells that were isolated by flow cytometry cell sorting. In this study, we examined whether single-cell isolated clones from CD44 low expressing cells could be induced by gemcitabine to express CD44. We sought to further determine the role, which may play in the tumorigenic phenotype of the cell. To mimic the clinical treatment, cells were treated transiently (16 h) with gemcitabine and then allowed to recover and the process was repeated weekly with increasing doses of the drug. Cells were collected and analyzed weekly.

**Fig. 1:**
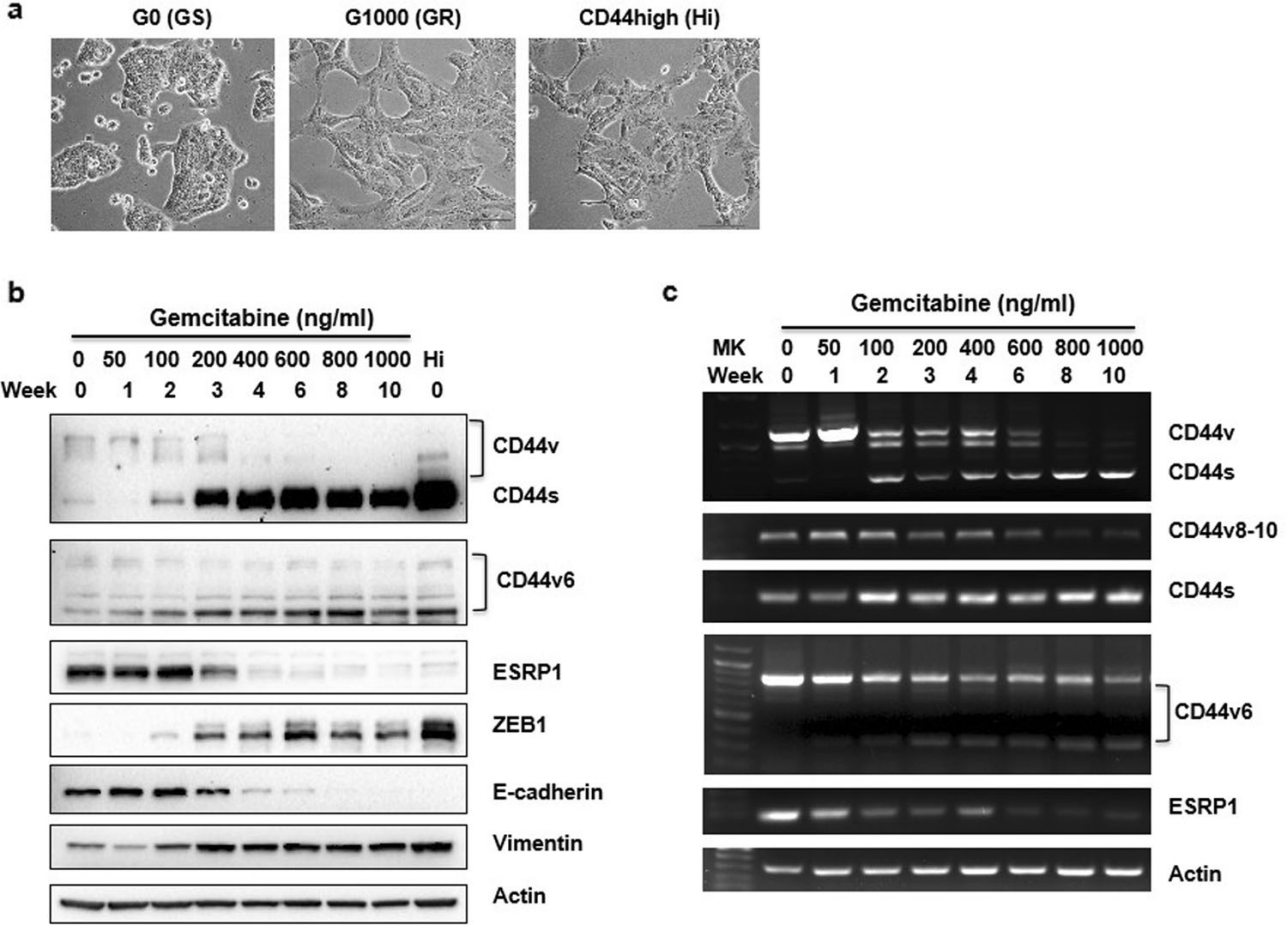
Gemcitabine treatment induces an EMT phenotype and CD44 expression with isoform switching. **a** Image of cell morphology was taken under the light microscope (×20 objective). **b**, **c** CFPAC1/GS cells were treated with gemcitabine overnight at various doses as indicated and recovered weekly for 2–3 months. Cells were analyzed for each dose and time of gemcitabine treatment. **b** Western blot analysis for the expression of CD44 isoforms and representative EMT marker proteins. **c** RT-PCR analysis of CD44 isoforms and ESRP1 at the different stages of gemcitabine treated cells.

After the treatment of CD44 low single cloned CFPAC1 and AsPC1 cells with elevated doses of gemcitabine weekly for 2–3 months, highly gemcitabine-resistant cells (GR) were obtained. CFPAC1 cells as a model were used for further studies. The GR cells displayed an EMT-like morphology, similar to CD44 high-expressing cells previously isolated by flow cytometry (Fig. 1a). An increase in CD44 expression and CD44 isoform switching from CD44v to CD44s was observed over time with increased concentrations of gemcitabine (Figs. 1b, c and S1a). The isoform switching was evidenced by the observation that higher molecular size CD44 variants v6 and v8–10 were diminished and lower sized or single variant exon v6 were increased along with a predominance of CD44s (Fig. 1b, c). These changes imitate the original CD44 high-expressing cells (Fig. 1b). We also noted that the expression of higher molecular weight CD44v that have multi-variant exons variants declined and single-variant exon variants increased and ESRP1, an epithelial splicing regulatory protein, was decreased with increasing doses of gemcitabine (Fig. 1b, c). Over the course of the treatment, an increase in ZEB1, a mesenchymal marker, and a loss of E-cadherin, an epithelial marker was observed and is consistent with conversion to EMT phenotype (Fig. 1b) and with the observed morphology (Fig. 1a). These findings support the premise that the development of drug-resistant clones is not solely the outgrowth of a drug-resistant cancer stem cell population but that drug-sensitive cells can be induced to switch to an EMT-like phenotype and cells that are more drug-resistant and invasive.

### CD44 confers drug resistance and invasiveness

**Fig. 2:**
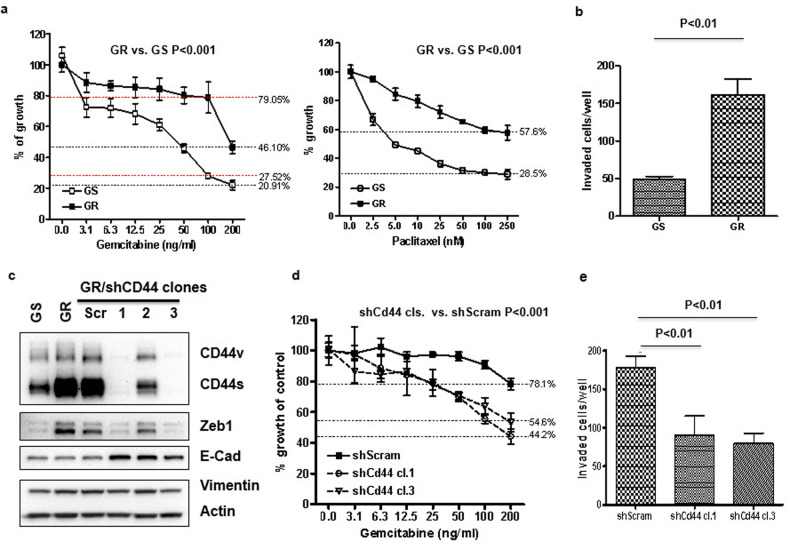
Gemcitabine-resistant cells show multi-drug resistance and are more invasive than their counterpart-sensitive cells. **a** Cell proliferation rate was measured for GR and GS cells treated with different doses of gemcitabine and paclitaxel for 3 days by MTT assays, the data were represented as mean ± SD in four replicates experiments. **b** 3 × 10^4^ cells/well of GS and GR cells were plated in the Matrigel invasion inserts (24-well size) for 24 h. Invaded cells were counted under the light microscope and the data were represented as mean ± SD performed in triplicate. **c** Western blot analysis of GR/shCD44 clones for the expression of CD44 and EMT makers. **d** GR and GR/shCD44 clones were treated with different doses of gemcitabine for 3 days, the cell proliferation rate was compared by MTT assays, and the data were represented as mean ± SD in four replicates experiment. **e** Matrigel invasion assay compared GR to GR/shCD44 cells, the data were represented as mean ± SD performed in triplicate.

To determine whether the drug resistance of GR cells was limited to gemcitabine, GR and GS cells were compared for sensitivity to gemcitabine and paclitaxel. Compared to GS cells, GR cells were more resistant to both gemcitabine and paclitaxel (Fig. 2a). GR cells were also more invasive (Fig. 2b). To examine the functional significance of CD44 in drug resistance and the invasion, we knocked down CD44 in GR cells using an shRNA against CD44 [36]. Single clones were selected and the efficacy of knocking down CD44 was confirmed by Western blotting (Fig. 2c). Two of three clones showed almost complete knockdown of CD44 (Fig. 2c, clones 1, 3), and these two clones were used for the following studies. Depletion of CD44 in GR cells suppressed the expression of ZEB1 and increased their expression of E-cadherin suggesting at least a partial reversal of EMT (Fig. 2c), increased their sensitivity to gemcitabine (Fig. 2d), and decreased their invasive potential as measured by Matrigel assays (Fig. 2e). It is noted that GR cells were maintained in culture for up to one month without adding gemcitabine but continued to show high expression of ZEB1and a subtle re-expression of E-cadherin (Fig. 1c).

### The development of gemcitabine resistance is associated with the induction of CD44 transcription

**Fig. 3:**
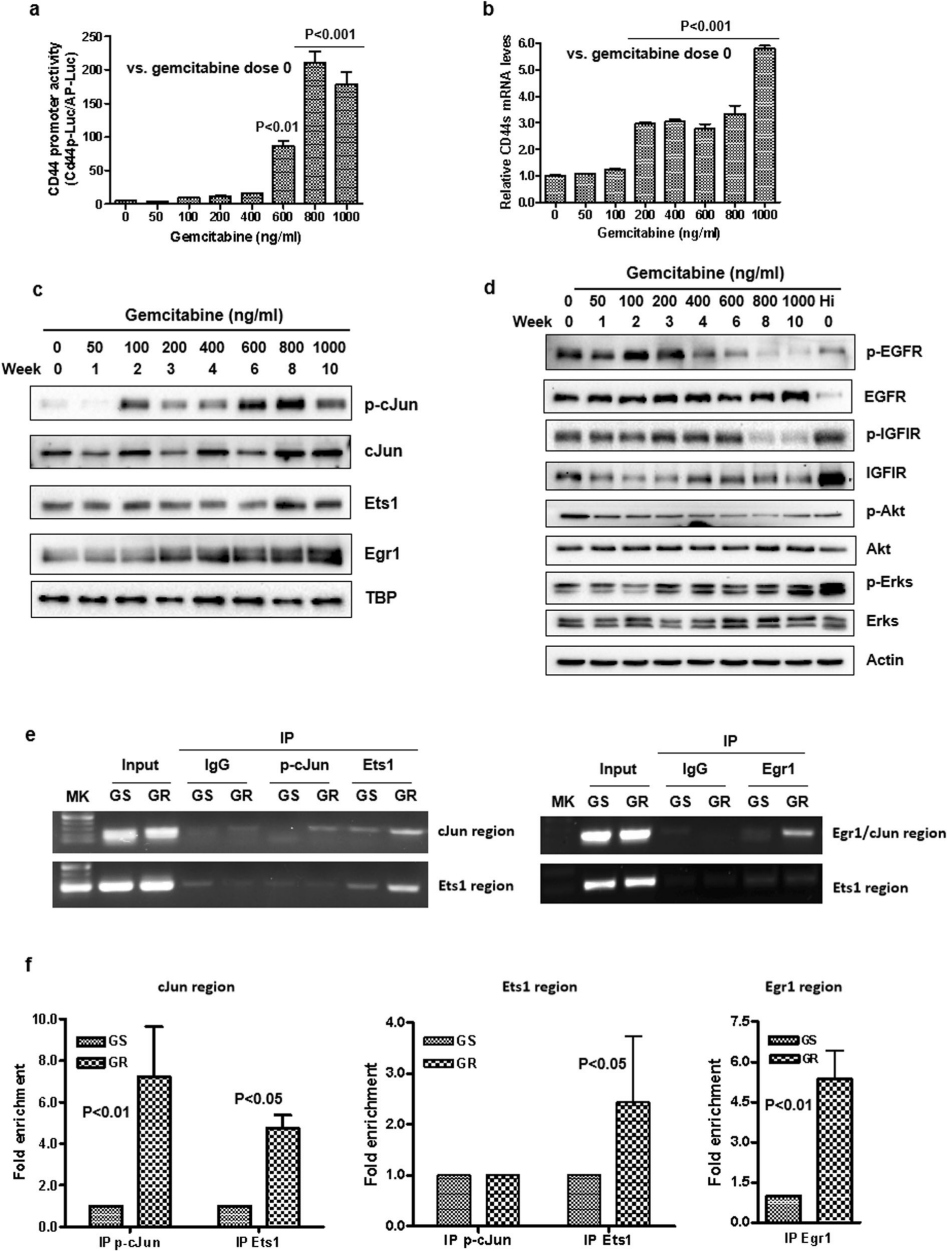
Induction of CD44 transcription and increase in expression of cJun, Ets1, and Egr1 during the development of gemcitabine-resistant cancer cells. **a** GS/CD44p-Luc cells were treated with elevated doses of gemcitabine weekly for up to 10 weeks. Conditioned media were collected for each treatment dose and assayed by a Secrete-Pair Dual Luminescence Assay Kit. The data were presented as mean ± SD in triplicate. *P* values were presented as compared to gemcitabine dose at 0 ng/ml. **b** Total RNA was isolated from cells as indicated in Fig. 1a and real-time RT-PCR was performed in triplicate for each dose using primers that only amplify CD44s. The relative CD44s expression was shown as mean ± SD. *P* values were presented as compared to gemcitabine dose at 0 ng/ml. **c** Nuclear proteins were isolated from the cells. The expression of transcriptional factors phosphor-cJun, cJun, Ets1, and Egr1 was detected by Western blot analysis. TBP was used as the loading control. **d** Western blot analysis of growth EGFR and IGF1R signaling in different dose stages of gemcitabine-treated cells. **e**, **f** Chromatin immunoprecipitation assays of binding of p-cJun, Ets1, and Egr1 to their regions of the CD44 promoter in GS and GR cells. **e** Samples were amplified by regular PCR, and the PCR products were run on 1.5% agarose gel. **f** Samples were analyzed by quantitative PCR. Fold enrichment is represented as signals obtained from immunoprecipitation with specific antibodies relative to signals obtained from immunoprecipitation with control IgG (mean ± SD from triplicate experiments).

Western blot and RT-PCR data suggested that gemcitabine-induced not only a CD44 isoform switch but also an increase in CD44 expression level with the CD44s as the predominant isoform (Fig. 1b, c). To determine the mechanism from the transcriptional perspective, CD44 promoter activity was measured during the course of gemcitabine treatments. Consistent with the protein level (Fig. 1b), the CD44 promoter activity and CD44s mRNA level gradually increased with the dose and duration of gemcitabine treatment (Figs. 3a, b and S3b). Previous studies indicated that the transcription factors c-Jun/AP1, Ets1, Egr1, and Sp1 directly bind to the CD44 promoter and are positive regulators of CD44 transcription [39, 41–43]. We extracted nuclear proteins from different times and doses of gemcitabine-treated cells analyzed by Western blotting. The results show that gemcitabine treatment induced a nuclear accumulation of phosphor-cJun, Ets1, and Egr1 (Figs. 3c and S1b). Also, a transient up-regulation in the phosphorylated forms of the growth factor receptors EGFR and IGF1R was observed (Fig. 3d). This finding suggests the possibility that activation of EGFR and IGF1R may be required for initiation of CD44 isoform switching and development of drug resistance, however, it may not be required for maintenance of the resistant phenotype. We applied chromatin-immunoprecipitation assays to explore whether these known transcriptional factors bound differently to the CD44 promoter during the transition of GS to GR cells. As shown in Fig. 3e, f, enhanced binding of these transcriptional factors to the CD44 promoter was observed in GR cells compared to GS cells by regular PCR (Fig. 3e) and by quantitative PCR (Fig. 3f). We also noted that Ets1 binding was elevated in the cJun region of CD44 promoter in GR cells, suggesting Ets1 and cJun may cooperate to activate CD44 transcription. An interaction of Egr1 and Ets1 was not observed.

### Early events following gemcitabine treatment show activation of IGF1R signaling and binding of transcriptional factors to CD44 promoter

**Fig. 4:**
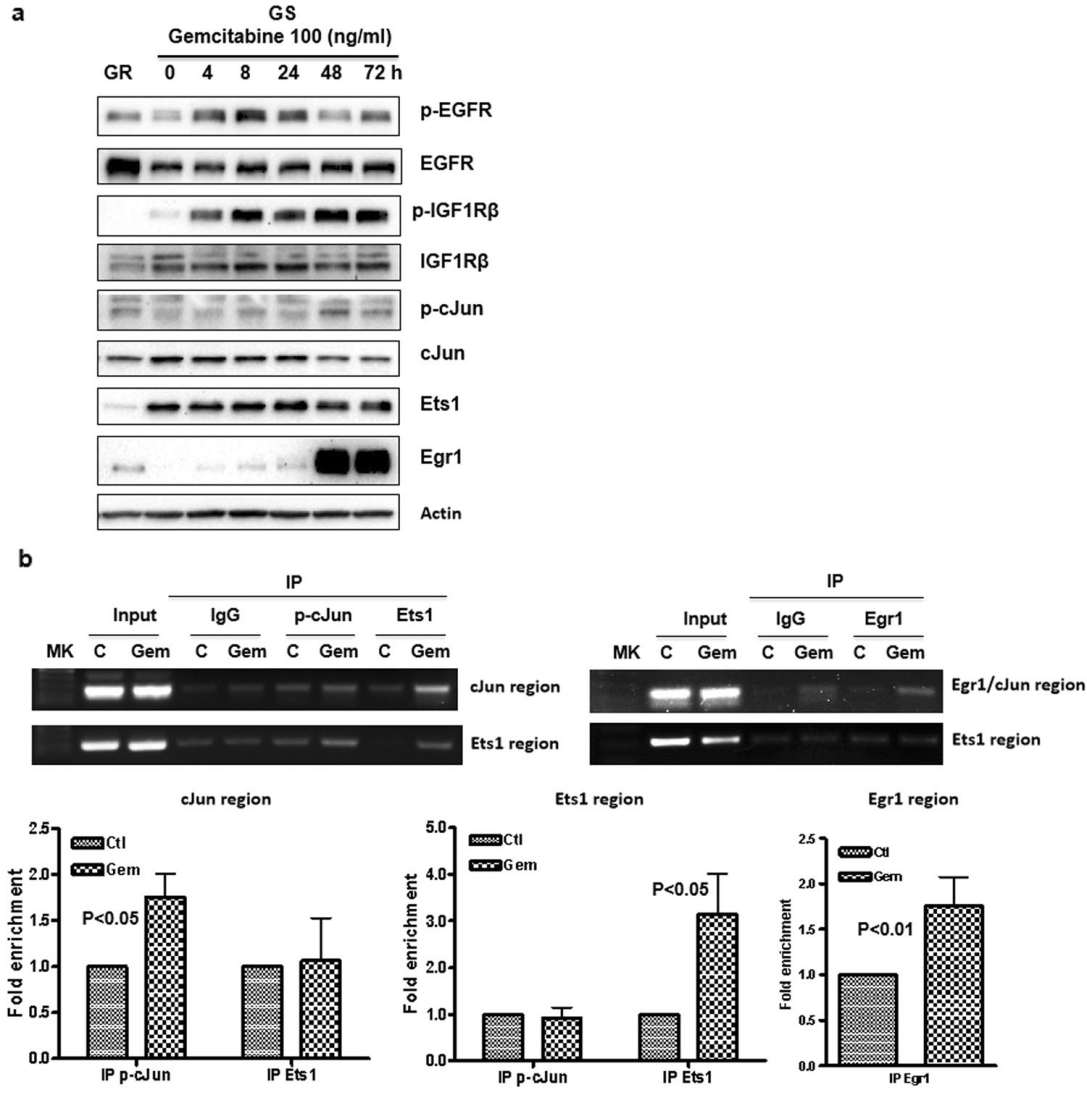
Gemcitabine treatment induces IGF1 signaling and binding of transcription factors p-cJun, Ets1, and Egr1 to CD44 promoter. **a** GS cells were treated with gemcitabine for the indicated period. EGFR, IGFR phosphorylation, and transcription factor p-cJun, Ets1, and Egr1 were detected by Western blot analysis. **b** GS cells were treated with gemcitabine for 48 h and chromatin immunoprecipitation assays were performed. The binding of p-cJun, Ets1, and Egr1 to CD44 promoter was analyzed by regular PCR (upper panel) and by quantitative PCR (lower panel). Fold enrichments are represented as signals obtained from immunoprecipitation with specific antibodies relative to signals obtained from immunoprecipitation with control IgG (mean ± SD from triplicate).

Stress from chemotherapy may induce changes in cell signaling that provide a survival advantage. These pathways may in turn activate transcriptional factors that contribute to the up-regulation of CD44 transcription and isoform switching. To determine this possibility, we examined whether EGFR and IGF1R were activated as an early event since they were activated in the first several weeks during long-term treatment with gemcitabine (Fig. 3d). We found that IGF1R expression and phosphorylation were markedly induced during the first 72 h of gemcitabine treatment (Fig. 4a). The activation of IGF1R begins at 4 h and last 72 h of the treatment. P-cJun, ETS1, and Egr1 were also transiently induced and Egr1 showed prolonged induction through 72-h treatment (Fig. 4a). To understand whether gemcitabine treatment induces these transcriptional factors binding to CD44 promoter, chromatin immunoprecipitation assay (ChIP) was performed. As shown in Fig. 4b, c, short-term gemcitabine-treated cells showed phosphor-cJun, Ets1, and Egr1 binding to the CD44 promoter (Fig. 4b). These findings suggest that gemcitabine rapidly induces IGF1R activation and binding of transcriptional factors to the CD44 promoter.

### IGF1R signaling mediates p-cJun, Ets1, and Egr1 binding and activation of CD44 promoter

**Fig. 5:**
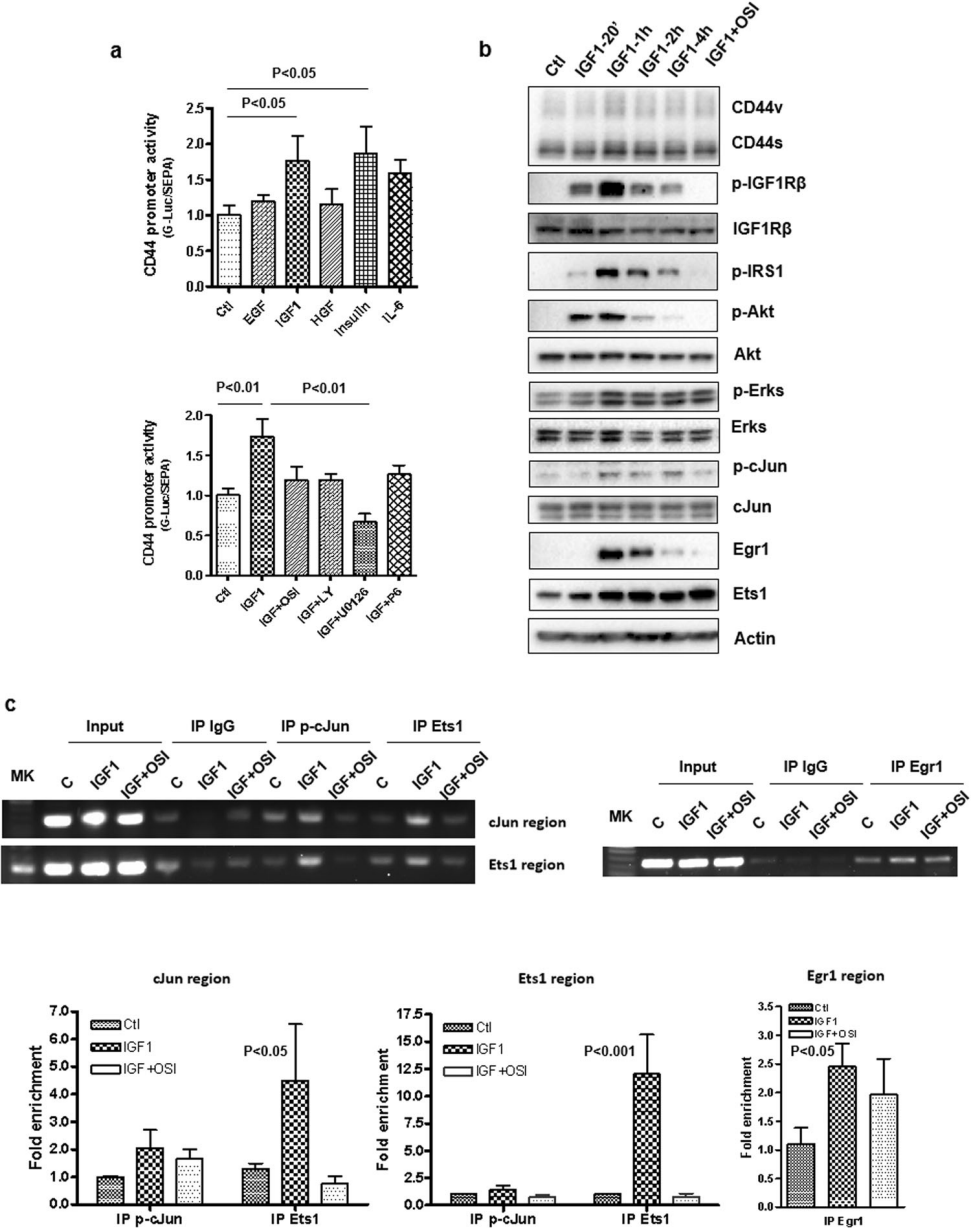
IGF1 signaling induces CD44 promoter activity and enhances p-cJun, Ets1, and Egr-1 binding to CD44 promoter. **a** 5 × 10^4^/well of GS/CD44p-Luc cells were plated in a 24-well plate and serum-starved overnight. Cells were than treated with inhibitors OSI 906 (100 nM), LY 294002 (20 μM), U0126 (10 μM) or pyridone 6 (P6, 5 μM) for 2 h followed by adding growth factors: EGF (10 ng/ml), IGF1 (20 ng/ml), HGF (10 ng/ml), and Insulin (20 ng/ml) for 48 h. Conditioned media were collected and luciferase activities and alkaline phosphatase activities were measured using Secrete-Pair Dual Luminescence Assay Kit according to the protocol. Data were presented as mean ± SD from triplicate experiments. **b** GS cells were serum-starved overnight and stimulated with recombinant 20 ng/ml IGF1 for the period as indicated. For the IGF1 inhibitor sample, OSI906 was added 2 h before adding IGF1 and cell lysate was harvested at the one-hour point of IGF1 addition. Western blot analysis was performed with indicated antibodies. **c** GS cells were treated with 20 ng/ml IGF1 or IGF1 plus inhibitor OSI 906 (100 nM) for 24 h. Chromatin immunoprecipitation assays with antibodies as indicated and normal IgG was used for non-specific binding control. Binding of p-cJun. ETs1 and Egr1 to CD44 promoter were shown in agarose gel with regular PCR products (upper panel) and quantitative PCR of ChIP samples (lower panel).

We next examined whether growth factor-mediated signaling activated the CD44 promoter. GS cells that were modified to stably express CD44 promoter-luciferase reporter were treated with a panel of growth factors. Both IGF1 and Insulin cytokines stimulated CD44 promoter activity (Fig. 5a, top panel). IGF1-induced CD44 promoter activity could be blocked by IGF1 signaling inhibitors, OSI-906, and PPP (Figs. 5a and S3b). Significant inhibition was seen with a MEK inhibitor U0126 and a PI3K inhibitor LY294002 (Fig. 5a, bottom panel). The effectiveness of these inhibitors to block their molecular targets was shown (Figs. S2 and S3c). To confirm whether IGF1R signaling enhanced CD44 promoter activity through induction of the above transcriptional factors, GS cells were treated with the recombinant human IGF1. The activation of IGF1R and nuclear accumulation of transcriptional factors phosphorylation of c-Jun, Egr1, and Ets1 were observed (Fig. 5b). ChIP assay determined whether IGF1 signaling increased these transcriptional factors binding to CD44 promoter. IGF1 stimulation increased the binding of phosphor-cJun, Ets1, and Egr1 to the CD44 promoter. One to two folds up-regulation of c-Jun binding to the c-Jun-binding site of the CD44 promoter was observed but was not statistically significant (Fig. 5c). The binding of Jun, Ets1, and Egr1 were blocked by IGF1R inhibitor OSI-906 (Fig. 5c), which suggested that IGF1 signaling promoting CD44 transcription may occur through activation of c-Jun, Ets1, and Egr1. Because of lacking statistical significance in c-Jun binding to the CD44 promoter, further molecular studies are required to determine whether it plays a role in mediating CD44 expression.

### Blocking IGF1R signaling prevents gemcitabine-induced CD44 isoform switching and EMT

**Fig. 6:**
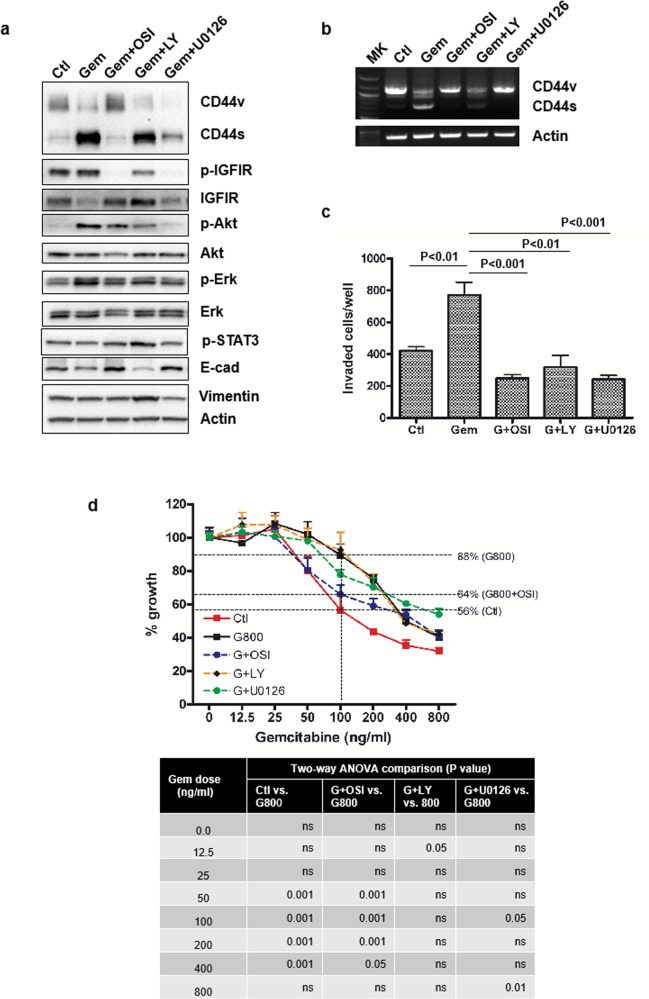
Blocking IGF1R signaling by pharmacological inhibitors prevents gemcitabine-induced CD44 isoform switching and EMT. **a** GS cells were treated with the doses of gemcitabine increased weekly (up to 800 ng/ml) and a constant dose of inhibitors (50 nM OSI-906; 10 μM LY294002; 5 μM U0126). Cell lysates were analyzed by Western blot with indicated antibodies. **b** RT-PCR analysis of CD44s and CD44v, primers were same as in Fig. 1c. **c** Invasion assay performed with the cells from the above treatment (gemcitabine 800 ng/ml). Invaded cells were stained and counted under the light microscope. Data were represented as mean ± SD performed in triplicate. **d** Cell proliferation rate was performed with the cells that survived from above treatments by MTT assay and the data were represented as mean ± SD in four replicates. The percentage of cell growth for each treatment compared to control at 100 ng/ml gemcitabine is presented. Statistical analysis by two-way ANOVA was shown in the table.

We next sought to determine whether blocking IGF1 signaling or downstream targets, MEK or PI3K could prevent gemcitabine-induced upregulation of CD44, CD44 isoform switching, EMT, invasiveness, and resistance to gemcitabine. Cells were continuously treated with inhibitors of IGF1R, MEK, or PI3K at concentrations determined to kill <20% of the cells and with increasing doses of gemcitabine up to 800 ng/ml over an 8-week period. At the end of 8 weeks, cells were analyzed by Western blotting and by RT-PCR. As observed before, treatment with gemcitabine alone caused an increase in CD44s expression with a decrease in expression of CD44v isoforms (Fig. 6a, b). Treatment with OSI-906, the IGF1R kinase inhibitor, prevented CD44 isoform switching and diminished the upregulation of CD44s expression and increased expression of E-cadherin, suggesting that this inhibitor prevented gemcitabine-induced EMT. The MEK inhibitors (U0126) partially blocked CD44 isoform switching (Fig. 6a). Interestingly, treatment with LY294002 in gemcitabine-treated cells appeared to potentiate EMT reflected by loss of E-cadherin and gain in Vimentin expression (Fig. 6a). All three inhibitors significantly reduced gemcitabine-mediated cell invasion (Fig. 6c). Gemcitabine-treated cells that were co-treated with OSI 906 showed a significant increase in sensitivity to gemcitabine (compared to gemcitabine dose at 100 ng/ml alone and cell growth rate of 800 ng/ml gemcitabine was from 88% to 64% of cells treated with gemcitabine and IGFR inhibitor OSI 906); whereas, treatment with U0126 or LY294002 showed less or none effect for enhancing drug sensitivities (Fig. 6d). A high dose of gemcitabine (800 ng/ml) continuously exposure for 3 days tremendously increased cell death in all treatment groups. Increased gemcitabine sensitivity in GR cells using a different IGF1R inhibitor, picropodophyllin which inhibited cell viability in the pancreatic cancer cells (Fig. S3a). These studies indicate that specific inhibitors particularly those that block IGF1R signaling may be useful in preventing gemcitabine-induced CD44 isoform switching and could be used to improve chemotherapeutic outcomes by blocking the development of CD44-dependent resistance and by inhibiting the invasive phenotype caused by gemcitabine treatment.

### Prognostic significance of CD44 and IGF1R expression in pancreatic cancer patients

The in vitro pancreatic cancer cell line data suggests that IGF1R signaling is required to induce a phenotypic switch of cells that possess low CD44 expression to one that shows a high level of CD44 expression with CD44s being the predominant isoform. This phenotypic switch to CD44s high cells results in cancer cells being more invasive and more resistant to chemotherapy. Based on these findings, the prognostic significance of CD44 and IGF1R expression in tumors from patients with pancreatic cancer were analyzed using the TCGA pancreatic cancer dataset. There were 178 patients with both CD44 and IGF1R expression, from which, the RNA expression data were divided into four groups: CD44-high/IGF1R-high, CD44-high/IGF1R-low, CD44-low/IGF1R-high, and CD44-low/IGF1R-low groups. These groups were compared for overall survival rate and 138 patients’ available data for determining progression-free survival rate.

**Fig. 7:**
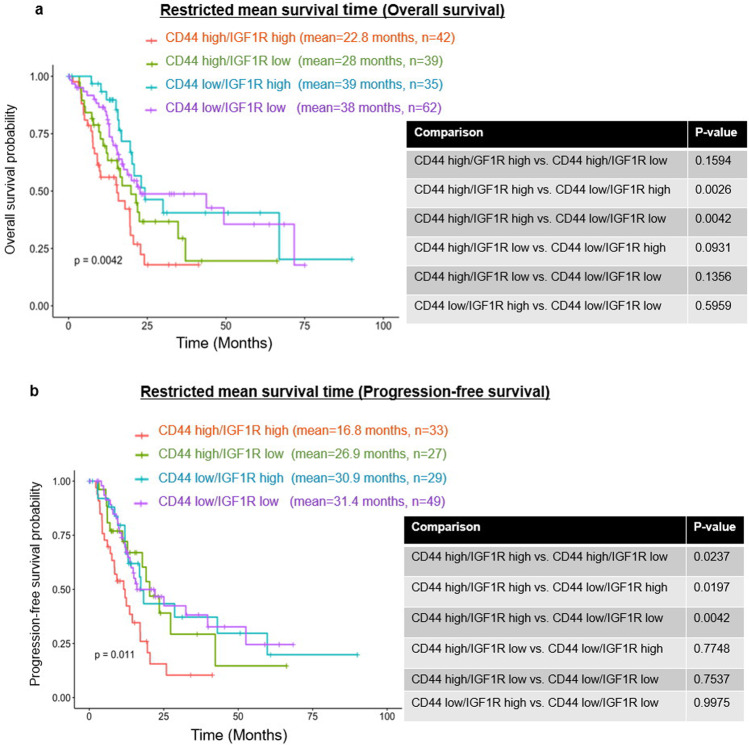
CD44 high/IGF1R high is significantly associated with poor prognosis in TCGA pancreatic cancer cohort. mRNA expression of CD44 and IGF1R extracted from cBioPortal TCGA_PAAD provisional cohort. Kaplan–Meier curve based on mRNA expression of CD44 and IGF1R was used to assess: **a** overall survival (*n* = 178) and **b** progression-free survival (*n* = 138).

The worst overall survival was seen in patients whose tumor was CD44-high/IGF1R-high (*n* = 42, mean survival 22.8 months) (Fig. 7a). Patients whose tumors were CD44-high/IGF1R-low (*n* = 39, mean survival 28 months) tended to have a slightly better prognosis than the CD44-high/IGF1R-high group although the difference was not statistically significant (Fig. 7a). These results are consistent with our current in vitro finding that the CD44-high phenotype is more tumorigenic and may require IGF1R signaling for initiation but not for the maintenance of this phenotype. CD44-low groups either IGF1R-high (*n* = 35, mean survival: 39 months) or IGF1R-low (*n* = 62, mean survival: 38 months) showed the longest overall survival (Fig. 7a). The worst progression-free survival was seen in the CD44-high/IGF1R-high group (*n* = 33, mean progression-free survival: 16.8 months), (Fig. 7b). Interestingly, the CD44-low/IGF1R-high group showed a significantly worst prognosis (*n* = 27, progression-free survival: 26.9 months) suggesting that high levels of IGF1R along with CD44-high expression were negatively correlated to progression-free survival (Fig. 7b). Patients with CD44-low expressing tumors showed the best progression-free survival times: CD44-low/IGF1R-high (*n* = 29, mean progression-free survival: 30.9 months) and CD44-low/IGF1R-low (*n* = 49, mean progression-free survival: 31.4 months) (Fig. 7b).

IGF1R expression levels were not an independent prognostic marker. As discussed above, the activation of IGF1R played a role in inducing CD44 promoter activity. This study suggests that patients with CD44-high expressing tumors have the worst prognosis and most likely have the poorest response to therapy. On the other hand, those patients whose tumors are CD44-low may be initially more responsive to chemotherapy and would be candidates for the new strategies for preventing phenotypic switching to CD44-high expressing tumors.

## Discussion

In this study, we examined the prognostic and functional significance of CD44 isoform switching and expression in PDAC. We previously showed using in vitro and in vivo models that CD44 high-expressing cells showed resistance to chemotherapy and an increase in invasiveness [21]. It was not clear whether the chemo-resistant phenotype was solely an outgrowth of CD44 high-expressing cells or whether PDAC cancer cells with an initial CD44 low phenotype can be induced by chemotherapy to undergo a phenotypic switch. We found that treating PDAC cells with gemcitabine induces CD44 low expressing cells to undergo an EMT and to become more invasive. These phenotypic changes were associated with an isoform switching of CD44 from expressing mainly low levels of CD44v to cells expressing high levels of CD44s. Cells with a CD44s high expressing phenotype show a selective advantage in survival and invasiveness in pancreatic cancer.

The interaction of CD44 and its ligand, hyaluronan, is complex and is thought to play a role in tumor progression [19]. Glycosylation of CD44 regulates its interaction with hyaluronan and intercellularly with signaling molecules [19, 25–27]. Moreover, CD44 variant isoforms are reported to be associated with the tumor metastasis [30, 31]. The present study does not address these possibilities but rather shows that chemotherapy-induced expression of CD44s mediates in part a chemo-resistant phenotype. It is possible that continuing phenotypic switching, including expression of exon variants from high CD44s expressing clones, may give rise to the tumor metastasis.

CD44 binds to a number of ligands which can cause conformational changes in the CD44 intracellular domain resulting in the binding of various signaling molecules [19]. The potential translocation of CD44 or its intracellular domain bound to signaling molecules has not been well studied. This possibility is plausible given that this occurs for other transmembrane receptors. Moreover, a recent study [44] indicates that CD44s upregulated in some tumor cells are found in extracellular vesicles although the functional significance of these vesicles was not established. The cellular localization of the increased CD44s found in GR-resistant cells may be of interest in future studies.

We further examined the molecular mechanism involved in the phenotypic switch of CD44 low cells to CD44 high expressing cells. One possibility is that gemcitabine may upregulate pathways that cause this switch in phenotype. Chemotherapy is known to induce compensatory activation of multiple growth factors mediated signaling pathways, such as EGFR and IGFR, and their downstream signaling PI3K/AKT and MEK/MAPK signaling [45–48]. Gemcitabine is reported to increase stem cell marker CD44 expression as well as apoptosis marker M30 [49]. CSCs are responsible for chemoresistance and inhibition of pancreatic cancer with CD44-positive cells that have antitumor effects [50]. CD44-positive pancreatic cancer cells were reported to be more resistant to gemcitabine as well [51]. These activated signaling pathways may promote cell survival and increase tumorigenic properties. We found that IGF1R was rapidly activated in response to gemcitabine. Increased levels of IGF1 in blood were found in multiple cancers, including colon, breast, prostate, lung cancer, and pancreatic cancer [52–54]. A high level of IGF1R in pancreatic cancer is associated with chemo-resistance, higher tumor grade, and decreased survival (Fig. 7 and [53, 55, 56]). Although, blocking IGF1R signaling by pharmaceutical inhibitors or blocking antibodies failed to show significant clinical benefit in pancreatic cancer [45, 57], a recent study by Camblin et al. showed that activation of IGF1R and ErbB3 reduced pancreatic cancer cells’ sensitivity to gemcitabine and paclitaxel and activation of ErbB receptor signaling confers resistance to IGF1R inhibition [45] in pancreatic cancer. Of note, IGF1R and ErbB3-bispecific antibodies enhance the efficacy of chemotherapy in patients with metastatic PDAC [58].

We demonstrated that IGF1R signaling was rapidly activated by treatment with gemcitabine and IGF1R played a role in regulating CD44 expression and isoform switching. Haojun Shi et al. demonstrated IGF1 drove CD44v6/C1QBP complex which phosphorylated IGF1R followed by activating its downstream PI3K and MAPK signaling pathways to promote the hepatic metastasis of pancreatic cancer cells [59]. However, once the cells become resistant to gemcitabine, phosphorylation of IGF1R is diminished (Fig. 3d), suggesting that chemo-resistant cells may not be dependent on IGF1R signaling for maintenance of the resistant phenotype. A recent study showed that CD44 isoform switching from expressing CD44v to CD44s was essential for epithelial to mesenchymal transition (EMT) during breast cancer progression [60]; whereas, CD44 variants are reported to be associated with metastatic lesions and with a poor prognosis [30, 61]. We demonstrated that activating IGF1R signaling by gemcitabine in the early stage induced CD44 isoform switching along with an EMT phenotypic change. These changes rendered cells more drug-resistant and more invasive. We also found that gemcitabine treatment up-regulated transcription factors, phosphor-cJun, Ets1, and Egr1 that induced CD44 promoter activity and promoted CD44 expression [39, 41, 62, 63]. Activation of IGF1R signaling upregulated the binding of cJun, Ets1, and Egr1 to CD44 promoters. These results suggest that blocking IGF1R signaling during gemcitabine treatment could prevent chemotherapy-induced CD44 expression or isoform switching and increase pancreatic cancer response to chemotherapy. To this point, we used IGF1R and its downstream signaling pathway inhibitors to understand whether any of them could prevent gemcitabine-induced CD44 isoform switching and EMT phenotype. We demonstrated that inhibiting IGFR and its downstream signaling pathway target MEK/Erk, but not PI3K/Akt in CD44 low cells prevented cells from gemcitabine-induced EMT and retained sensitivity to gemcitabine. This finding suggests that activation of IGFR and the MEK/Erk pathway plays an important role in developing chemo-resistance. Interestingly, inhibiting of PI3K/Akt pathway caused cells to become more EMT-like and resistant to gemcitabine, but less invasive, indicating that the resistant cells did not rely on PI3K/Akt pathway for survival; however, these cells could represent a subpopulation of inactive stem-like cells. Although both MEK/Erk and PI3K/Akt are downstream targets of the IGFR signaling pathway, they play different roles in mediating biology processes and cell function. MEK/Erk and PI3K/Akt could also be activated by different growth factors, such as EGFR and ErbB3 [45–47], thus tumor types and stages, as well as CD44 isoforms and expression levels should be considered when these inhibitors are combined with chemotherapy for PDAC treatment.

In summary, the study here indicates that gemcitabine can induce pancreatic cancer cells to undergo a phenotypic shift involving the upregulation in the expression of CD44s isoform accompanied by EMT. Similar results had been observed in paclitaxel-resistant gastric cancer cells that paclitaxel enriched CD44 population accompanied with EMT [64]. Cancer cells undergoing this phenotypic switch show an increase in resistance to chemotherapy and are more invasive. These findings may help explain the molecular basis of why pancreatic cancer patients initially respond to chemotherapy generally but gradually develop drug resistance during the treatment. Early events in this switch involve the upregulation of IGF1-R-dependent transcription factor binding to the CD44 promoter. Our study also opened a new field that chemotherapy such as gemcitabine [65] or paclitaxel tends to enrich CD44-positive cells, especially CD44s isoform in various cancer types [64, 66]. Therefore, any long-term chemotherapy which may drive CD44 isoforms expression or switching should be under caution avoiding inducing CD44 upregulation at an early time. Our results suggest that PDAC patients with low CD44 expression tumors may be amenable to combined gemcitabine and inhibitors of IGFR or its downstream molecular targets. The strategy could prevent the development of CD44 high/chemo-resistance tumor cells and improve patients’ survival rates.

## Materials and methods

### Cell cultures and reagents

Human pancreatic ductal adenocarcinomas (PDAC) cell lines CFPAC-1 and AsPC-1 were from ATCC. Cells were maintained in the medium as recommended by ATCC and supplemented with 10% FBS in a 37 °C, 5% CO_2_ incubator. The plasmid shCD44-2 pRRL which targets all isoforms of human CD44 was a gift from Bob Weinberg (Addgene plasmid #19123; http://n2t.net/addgene:19123;) [36]. pEZX-LvPG04--CD44 promoter-Gluc plasmid was purchased from GeneCopoeia (Rockville, MD). The plasmids were transfected into 293T packaging cells (ATCC) using FuGENE 6 (Roche Applied Science) according to the manufacturer’s protocol. The pancreatic cancer cells were then infected with the viral medium collected from the packaging cells 48 h after the transfection. The CD44 knockdown cells were selected by GFP cell sorting (Flow Cytometry Core of the University of Texas Health Science Center at San Antonio, TX) followed by the limited dilution for the selection of single-cell clones. CD44 protein level was determined by Western blotting analysis. For generating CD44 promoter Gaussia luciferase-expressing cells, the cells were selected with 1 μg/ml of puromycin, and the expression of Gaussia-luciferase was determined using Secrete-Pair Dual Luminescence Assay Kit (GeneCopoeia, Rockville, MD). Gemcitabine hydrochloride was purchased from Abcam Biochemicals (Cambridge, UK) and was dissolved in the sterile saline solution. Reverse transcription reagents and real-time PCR SYBR Green Supermix were purchased from Biorad (Hercules, CA). Growth factors EGF, IGF1, HGF, and IL-6 were from R&D (Minneapolis, MN). Insulin was obtained from Sigma-Aldrich (St. Louis, MO). Pharmaceutical inhibitor, OSI-906 was from SelleckChem (Huston, TX); LY294002, pyridone 6 (P6), and U0126 were from Calbiochem (Burlington, MA), picropodophyllin (PPP) was from MedChemExpress (NJ, USA).

### Development of gemcitabine-resistant cell lines

The human pancreatic adenocarcinoma cell line CFPAC-1 and AsPC-1 were obtained from ATCC and were cultured in RPMI or DMEM media supplemented with 10% fetal bovine serum. These PDAC cell lines were single-cloned and screened by Western blotting for CD44 expression. To develop gemcitabine-resistant cells, CD44 low single clone cells were transiently exposed to gemcitabine for 16 h once a week with increasing concentrations (50 ng/ml to 1.0 µg/ml) of gemcitabine weekly for more than 2 months. The resulting gemcitabine-resistant cells were referred to as GR and the original gemcitabine-sensitive cells were referred to as GS.

### Western blots analyses

Western blot analysis was performed as described previously [37]. Primary antibodies used were as follows: E-cadherin, phos-cJun and cJun, Ets1 were purchased from Santa Cruz Biotechnology Inc. (Santa Cruz, CA); CD44, ZEB1, Egr-1, phos-IGF1Rβ, IGF1Rβ, phos-EGFR, and EGFR were purchased from Cell Signaling Technology (Beverly, MA); Vimentin was from Life Technologies (Carlsbad, CA). Horseradish peroxidase-conjugated secondary antibodies were purchased from Amersham Biosciences (Piscataway, NJ).

### Matrigel invasion assays

Cell invasiveness was analyzed by Matrigel invasion assays as described elsewhere [37]. Briefly, cells (3 × 10^4^ per well) were plated in 24-well Matrigel invasion chambers (Becton Dickinson Labware) in 0.5 ml of serum-free medium. The outer chambers contained 0.7 ml of medium containing 10% fetal bovine serum. The cells were incubated for 24 h. The cells seeded on the inner surface of the membrane were gently removed with cotton swabs. The cells migrating to the undersurface of the membrane were fixed in 70% ethanol and stained with 0.1% crystal violet. The invaded cells were counted under the light microscope (×10).

### Luciferase assay to measure CD44 promoter activity

CD44 promoter Gaussian-luciferase and secreted alkaline phosphatase were stably expressed in GS cells (GS/CD44p-Luc) and treated with weekly increased doses of gemcitabine as described in the paragraph on the development of gemcitabine resistant cell lines. For growth factor stimulation and pathway inhibitor treatment, GS/CD44p-Luc cells were serum-starved overnight, then treated with different pathway inhibitors 2 h before adding growth factors. Conditioned media were collected 48 h after plating cells and CD44 promoter activity was determined using Secrete-Pair Dual Luminescence Assay Kit accordingly.

### Quantitative real-time polymerase chain reaction (qRT-PCR)

Total RNA was isolated from cultured cells by TRIzol reagent (Invitrogen) and 1.0 μg of total RNA was reverse transcribed using iScript Select cDNA Synthesis kit (BioRad). 50 ng of cDNA was used for the following PCR reaction. PCR primers used for amplifying CD44s, CD44v6, and CD44v8-10 have been described previously [38]. The quantitative PCR reaction was done with iQ SYBR Green Supermix (Bio-Rad) according to the manufacturer’s instructions.

### Chromatin immunoprecipitation (ChIP) Assays

Chromatin immunoprecipitation was performed using ChIP-IT Express Kits from Active Motif (Carlsbad, CA). Briefly, cells were fixed in 1% formaldehyde and lysed. Chromatin was sheared to the size of around 300–1000 bp fragments. 20 μg of chromatin was subjected to immunoprecipitation using specific antibodies. The binding of transcriptional factor to the CD44 promoter was determined by regular PCR and quantitative PCR using the specific binding region primers corresponding to the CD44 promoter. Primers used are 5’-cagcgggagaagaaagccag-3’ (forward) and 5’-agtgacctaagacggagggag-3’ (reverse) for cJun site; 5’-gaacgtatgggtggatgagag-3’ (forward) and 5’-caaccacctattcttctattc-3’ (reverse) for Ets1 site [39] and 5’-gttcggtcatcctctgtcctg-3’ (forward) and 5’-gagcgaaggacacacccaag-3’ (reverse) for Egr1 site. The 2^−ΔΔct^ method was used for calculating the relative binding capacity of specific transcriptional factors.

### Analysis of the clinical relevance of CD44 and IGF1R expression in human pancreatic adenocarcinoma tissues

Z-transformed RNA-Seq data were downloaded from The Cancer Genome Atlas (TCGA) PAAD provisional cohort (http://www.cbioportal.org) [40]. Mean value was used to separate samples into the high and low expressions for CD44 and IGF1R. Kaplan–Meier curve was created using the R package with *P*-value determined by a log-rank test.

### Statistical analysis

All the experiments were at least performed independently three times. Statistical analysis was performed using GraphPad InStat software (GraphPad Software, Inc.). The significance of differences among groups was determined by Student *t*-test and one-way or two-way ANOVA followed by Bonferroni correction for multiple comparison tests accordingly. *P*-value < 0.05 was considered statistically significant.

## Supplementary information

Original Data File figure 1-6

Supplemental figures and suppl. figure legends

aj-checklist

## Data Availability

All data and information concerning this study will be made available upon request.
