# Supplementary material for: Gemcitabine resistance of pancreatic cancer cells is mediated by IGF1R dependent upregulation of CD44 expression and isoform switching
Source: Cell Death Dis. 2022 Aug 5;13(8):682. doi: 10.1038/s41419-022-05103-1 (PMC9355957; doi:10.1038/s41419-022-05103-1)

Fig. 1b

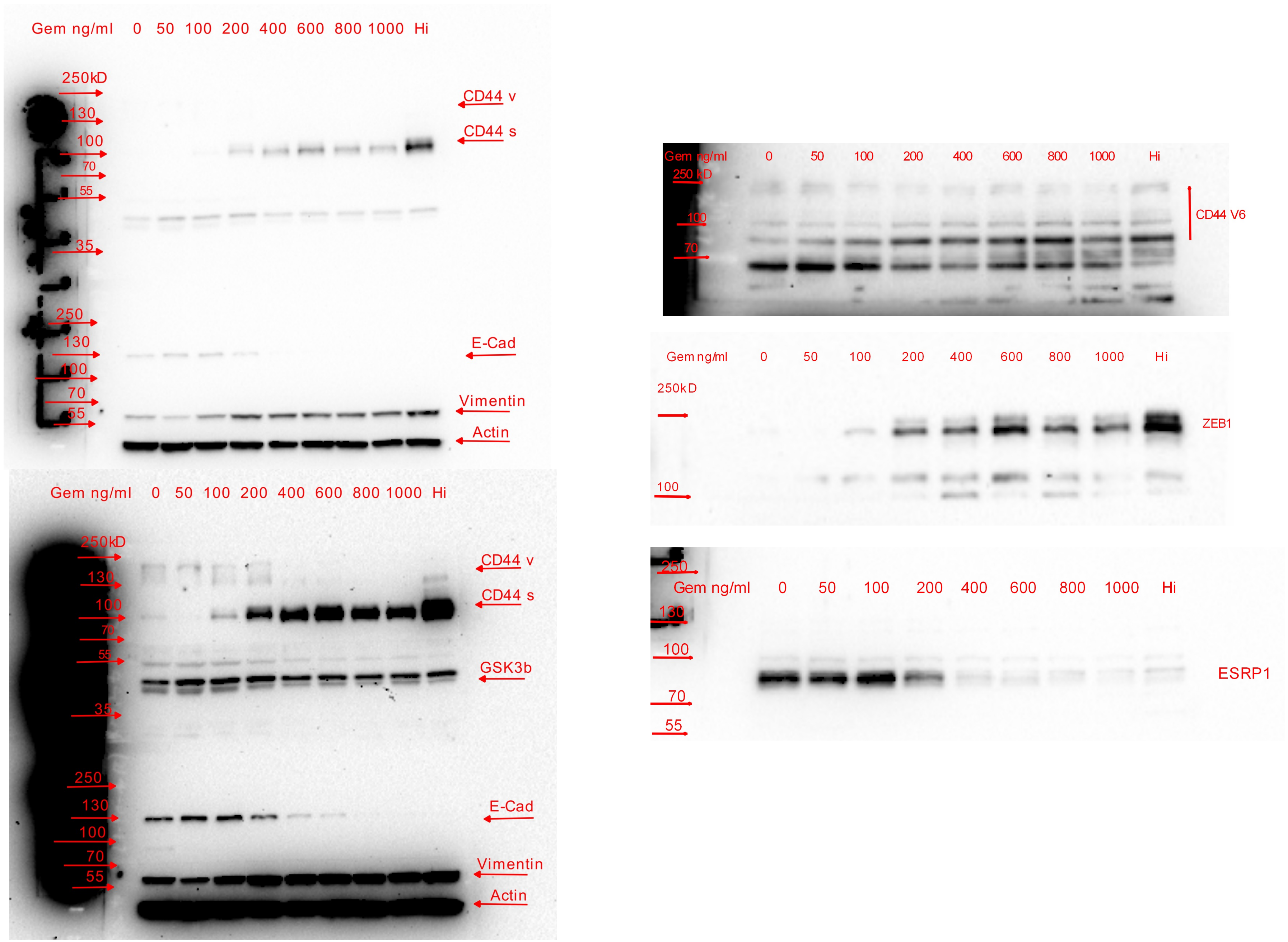
Gem ng/ml 0 50 100 200 400 600 800 1000 Hi
250kD
130
100
70
55
35
250
130
100
70
55
CD44 v
CD44 s
E-Cad
Vimentin
Actin
Gem ng/ml 0 50 100 200 400 600 800 1000 Hi
250kD
130
100
70
55
35
250
130
100
70
55
CD44 v
CD44 s
GSK3b
E-Cad
Vimentin
Actin
Gem ng/ml 0 50 100 200 400 600 800 1000 Hi
250 kD
100
70
CD44 V6
Gem ng/ml 0 50 100 200 400 600 800 1000 Hi
250kD
100
ZEB1
250
Gem ng/ml 0 50 100 200 400 600 800 1000 Hi
130
100
70
55
ESRP1

Fig. 2c

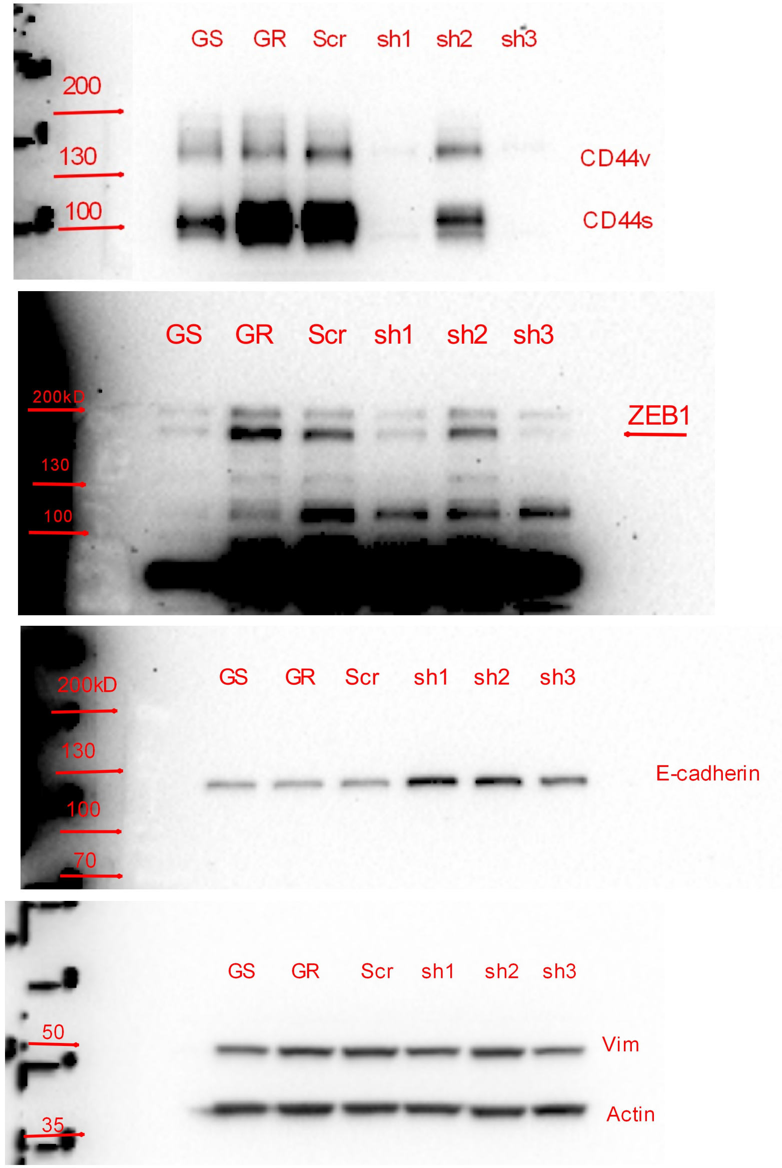
GS GR Scr sh1 sh2 sh3
200
130
100
CD44v
CD44s
GS GR Scr sh1 sh2 sh3
200kD
130
100
ZEB1
GS GR Scr sh1 sh2 sh3
200kD
130
100
70
E-cadherin
GS GR Scr sh1 sh2 sh3
50
35
Vim
Actin

Fig. 3c

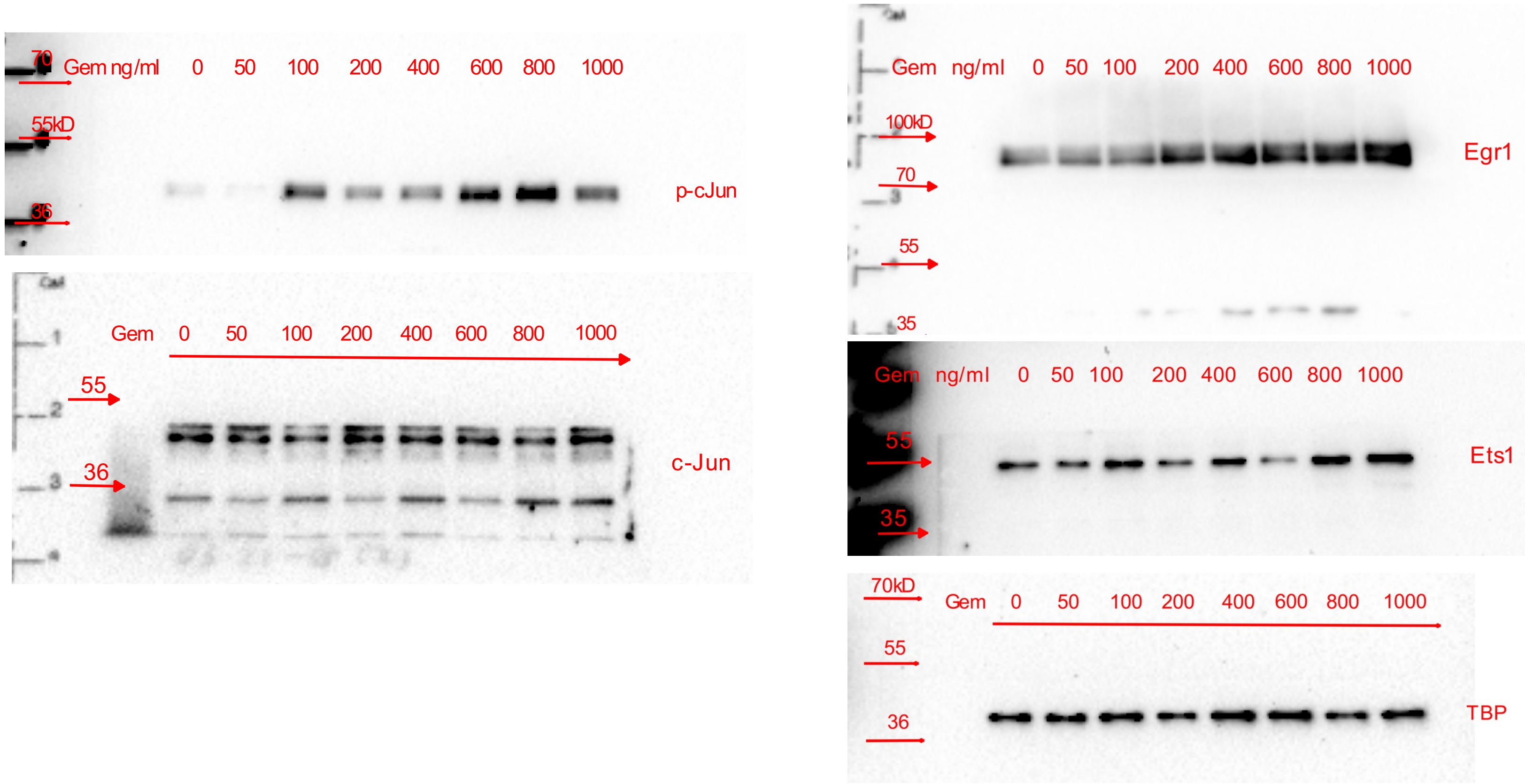

Gem ng/ml 0 50 100 200 400 600 800 1000
70
55kD
36
p-cJun
Gem 0 50 100 200 400 600 800 1000
55
36
c-Jun
Gem ng/ml 0 50 100 200 400 600 800 1000
100kD
70
55
35
Egr1
Gem ng/ml 0 50 100 200 400 600 800 1000
55
35
Ets1
70kD
Gem 0 50 100 200 400 600 800 1000
55
36
TBP

Fig. 3d

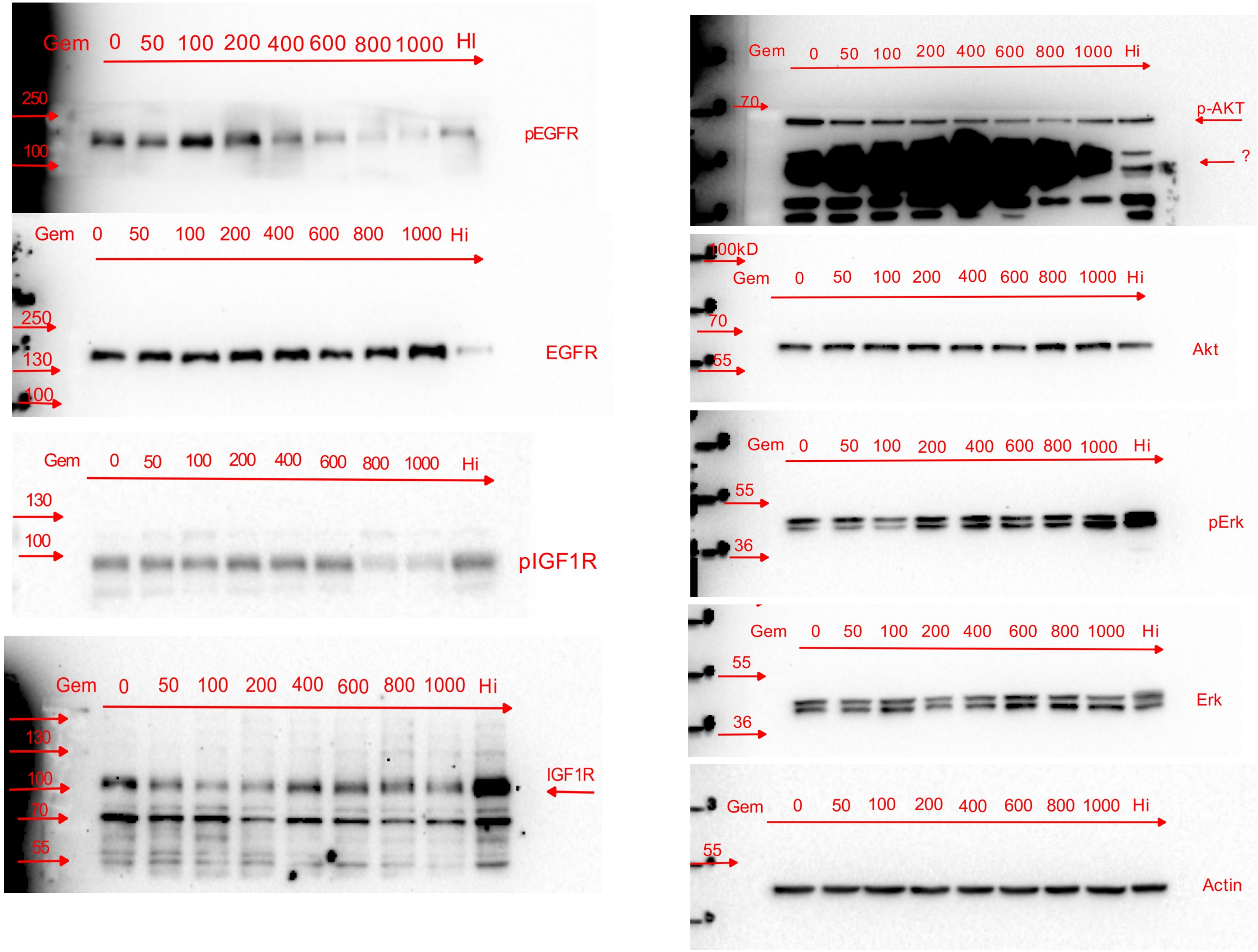
Gem 0 50 100 200 400 600 800 1000 HI
250
100
pEGFR
Gem 0 50 100 200 400 600 800 1000 Hi
250
130
100
EGFR
Gem 0 50 100 200 400 600 800 1000 Hi
130
100
pIGF1R
Gem 0 50 100 200 400 600 800 1000 Hi
130
100
70
55
IGF1R
Gem 0 50 100 200 400 600 800 1000 Hi
70
p-AKT
?
100kD
Gem 0 50 100 200 400 600 800 1000 Hi
70
55
Akt
Gem 0 50 100 200 400 600 800 1000 Hi
55
36
pErk
Gem 0 50 100 200 400 600 800 1000 Hi
55
36
Erk
Gem 0 50 100 200 400 600 800 1000 Hi
55
Actin

Fig. 4a

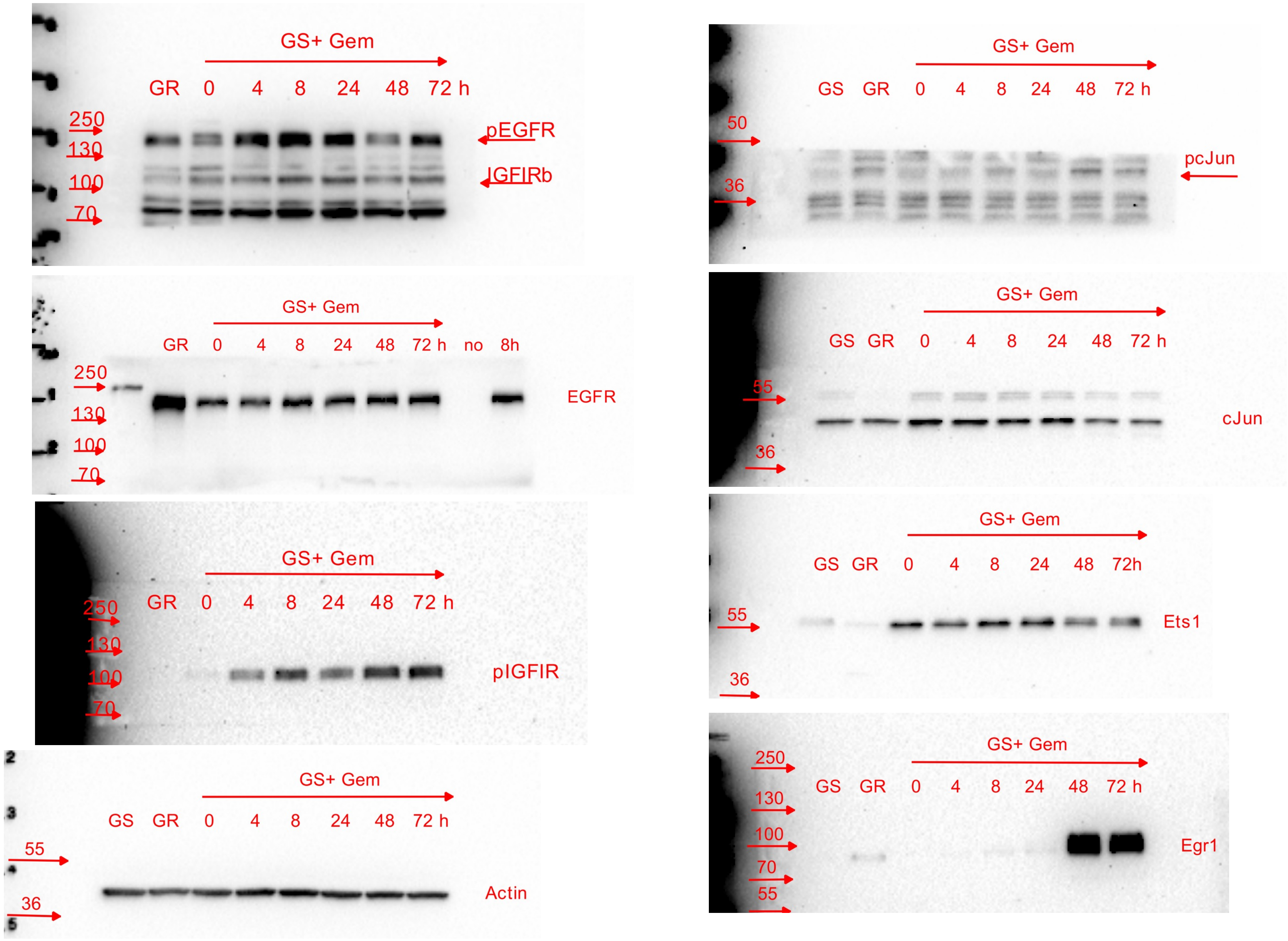
GS+ Gem
GR 0 4 8 24 48 72 h
250
130
100
70
pEGFR
IGFIRb
GS+ Gem
GR 0 4 8 24 48 72 h no 8h
250
130
100
70
EGFR
GS+ Gem
GR 0 4 8 24 48 72 h
250
130
100
70
pIGFIR
GS+ Gem
GS GR 0 4 8 24 48 72 h
55
36
Actin
GS+ Gem
GS GR 0 4 8 24 48 72 h
50
36
pcJun
GS+ Gem
GS GR 0 4 8 24 48 72 h
55
36
cJun
GS+ Gem
GS GR 0 4 8 24 48 72h
55
36
Ets1
GS+ Gem
GS GR 0 4 8 24 48 72 h
250
130
100
70
55
Egr1

Fig. 5b (dash line left part GS cells)

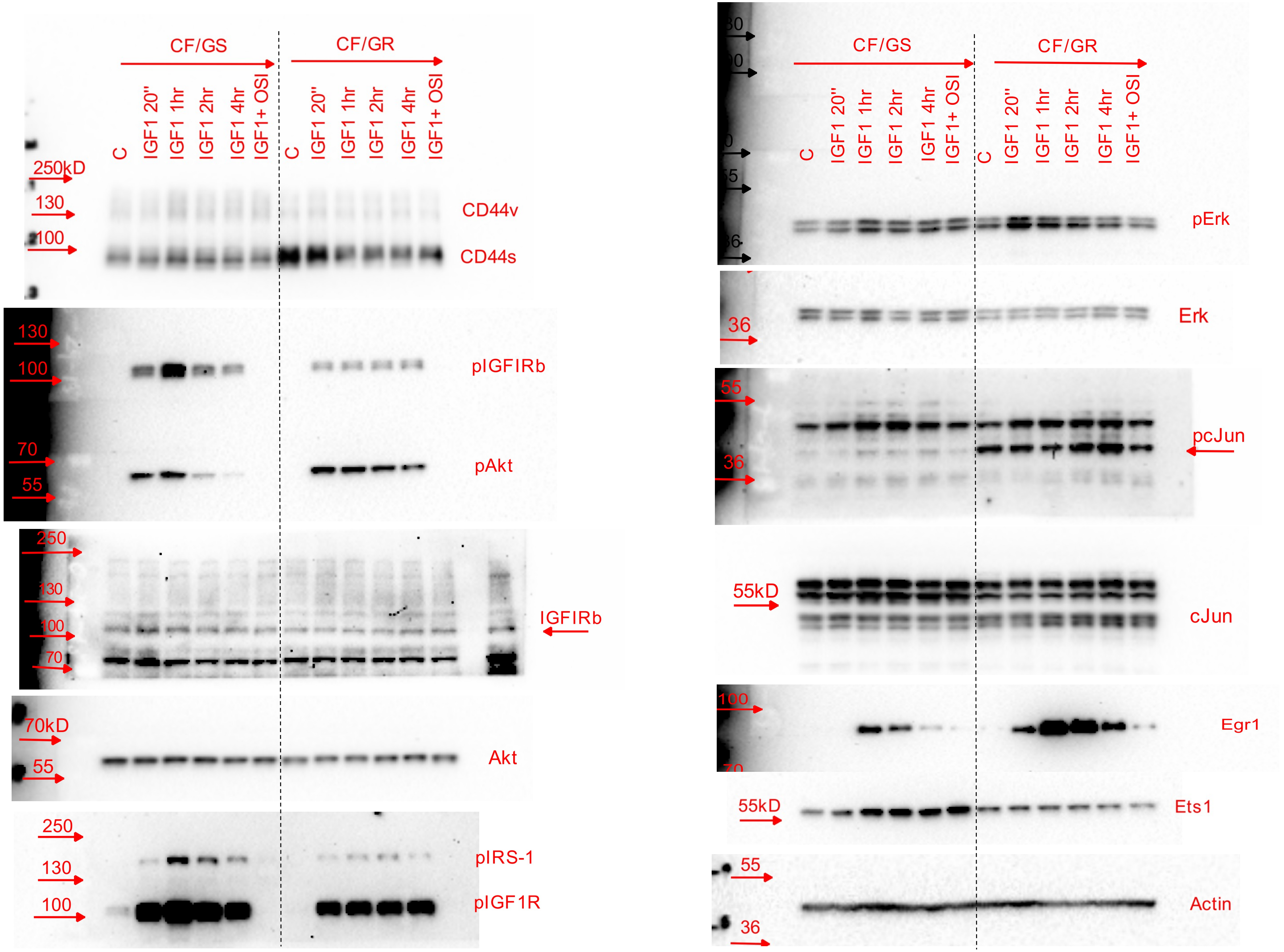

Fig. 6a (dash line left part and right panel)

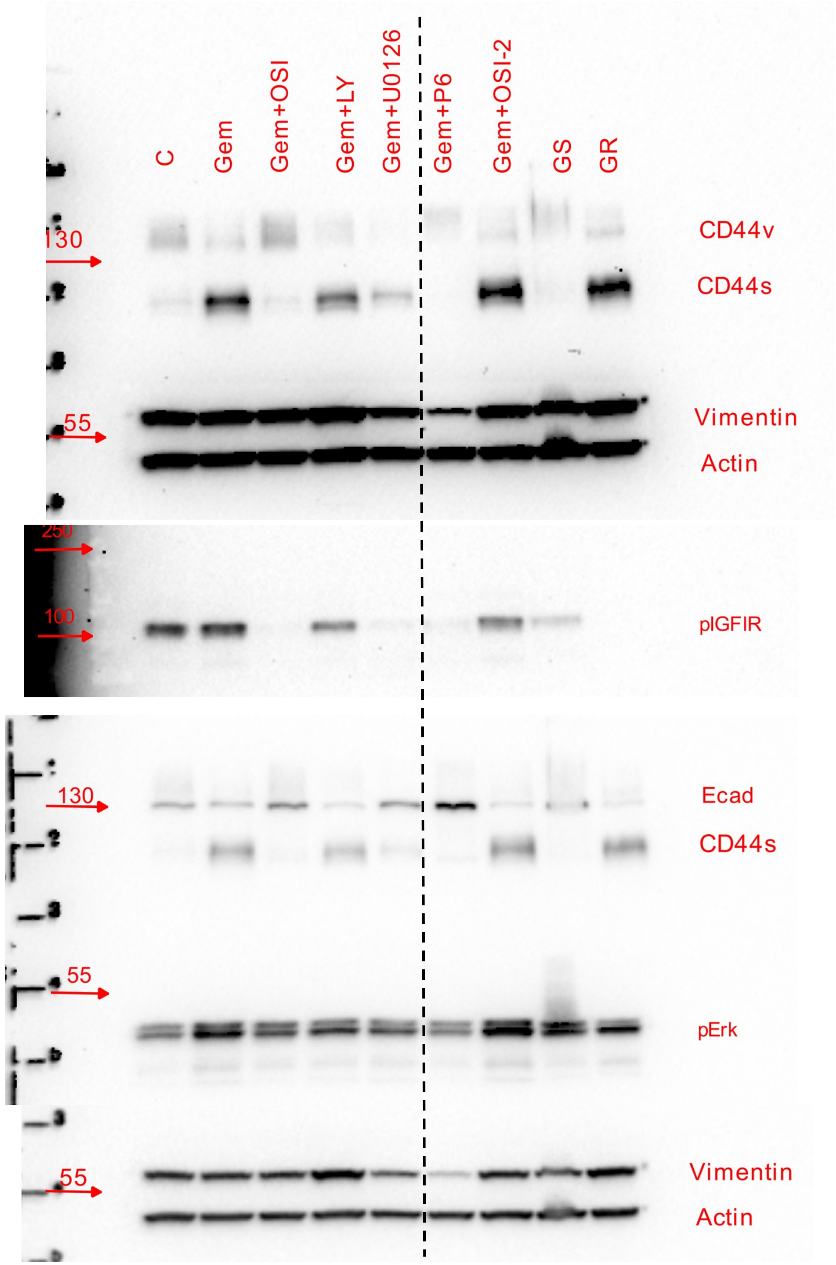

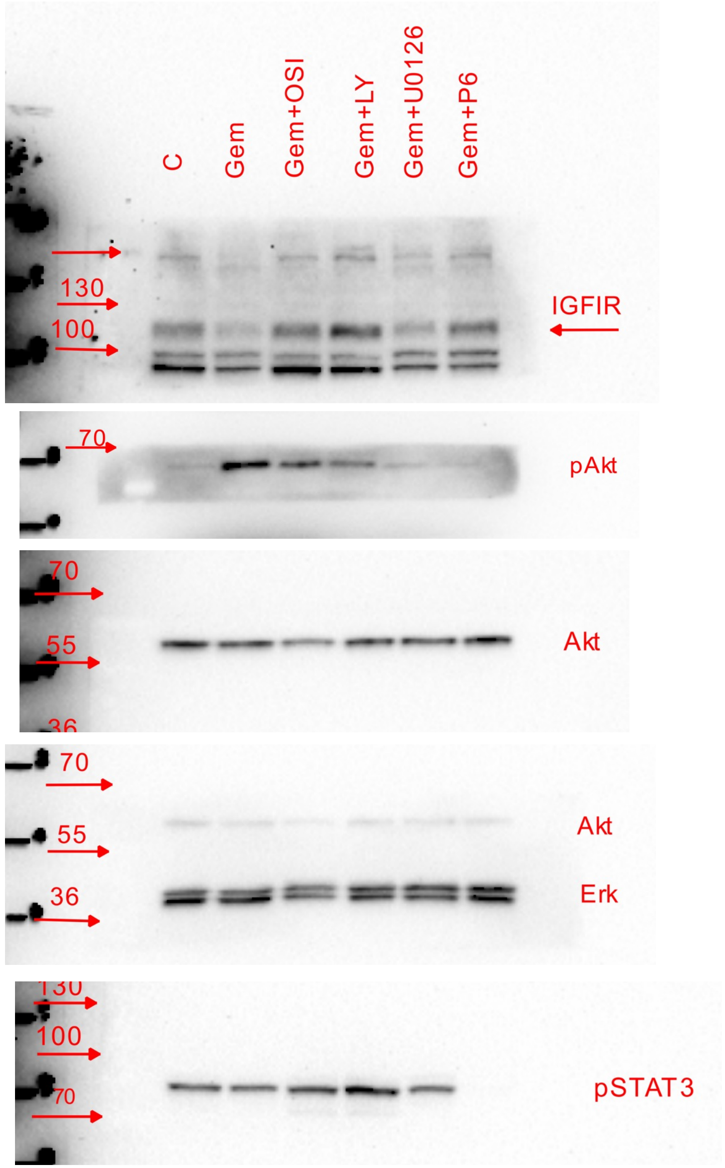

Supplement: Supplementary file 1 — Original Data File figure 1-6 [file 41419_2022_5103_MOESM1_ESM.pdf]
