# Supplementary material for: Gemcitabine resistance of pancreatic cancer cells is mediated by IGF1R dependent upregulation of CD44 expression and isoform switching
Source: Cell Death Dis. 2022 Aug 5;13(8):682. doi: 10.1038/s41419-022-05103-1 (PMC9355957; doi:10.1038/s41419-022-05103-1)

Supplemental Fig.1 **Gemcitabine treatment induced an EMT phenotype and CD44 expression with an isoform switching in AsPC1/GS cells**. AsPC1/GS cells were treated with elevated doses of gemcitabine weekly as indicated. **a** Western blot analysis for the expression of CD44 isoforms and EMT marker proteins. **b** Western blot analysis for the expression of p-IGF1R and transcriptional factors.

Supplemental Fig. 2 **Pathway inhibitors blocked IGF1-induced activation of their molecular targets.** GS cells were serum-starved overnight and treated with different pathway inhibitors (50 nM OSI906; 10 μM LY294002; 5 μM U0126 for 2 hours, then stimulated with 20 ng/ml IGF1 for another one hour. Cell lysates were analyzed by Western blot with antibodies as indicated. Inhibitors blocked the IGF1 induced phosphorylation of its downstream molecules is shown.

Supplemental Fig. 3 **Inhibition of IGF1R signaling by PPP sensitized GR cells to gemcitabine and blocked IGF1 induced CD44 promoter activity.** **a.** Cell proliferation rate was measured by MTT assays in GR and GS cells treated with gemcitabine and the combination of gemcitabine and different IGF1R inhibitors for 3 days. The data were represented as mean ± SD in 6 replicates experiments. **b**. GS/CD44p-Luc cells (5x10^4^/well) were plated in a 24 well plate and serum-starved overnight. Cells were then pre-treated with inhibitors OSI 906 (100nM) or PPP (5μM) for 2 hours followed by adding IGF1 (20 ng/ml) for 48 hours. Conditioned media were collected and luciferase activities and alkaline phosphatase activities were measured using Secrete-Pair Dual Luminescence Assay Kit according to the protocol. Data were presented as mean ± SD from triplicate experiments. **c.** GS cells were serum-starved and pre-treated with IGF1R inhibitor PPP overnight then stimulated with IGF1 (20 ng/ml) for 1 hour. Cell lysates were analyzed by Western blot with indicated antibodies.


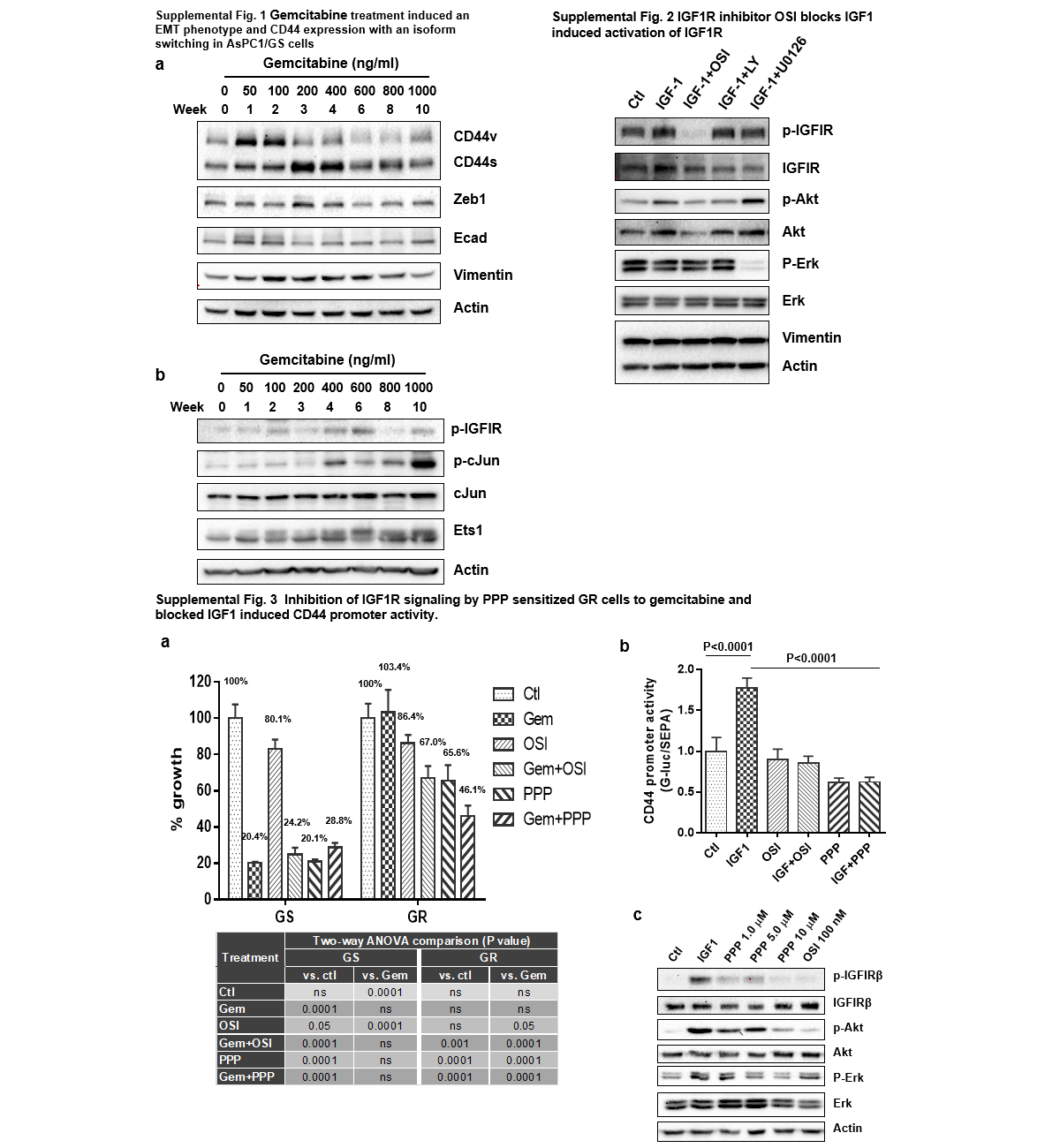

Supplement: Supplementary file 2 — Supplemental figures and suppl. figure legends [file 41419_2022_5103_MOESM2_ESM.docx]
